# Reptin Regulates DNA Double Strand Breaks Repair in Human Hepatocellular Carcinoma

**DOI:** 10.1371/journal.pone.0123333

**Published:** 2015-04-15

**Authors:** Anne-Aurélie Raymond, Samira Benhamouche, Véronique Neaud, Julie Di Martino, Joaquim Javary, Jean Rosenbaum

**Affiliations:** 1 INSERM, U1053, F-33076 Bordeaux, France; 2 Université de Bordeaux, F 33076, Bordeaux, France; Dana-Farber/Harvard Cancer Institute, UNITED STATES

## Abstract

Reptin/RUVBL2 is overexpressed in most hepatocellular carcinomas and is required for the growth and viability of HCC cells. Reptin is involved in several chromatin remodeling complexes, some of which are involved in the detection and repair of DNA damage, but data on Reptin involvement in the repair of DNA damage are scarce and contradictory. Our objective was to study the effects of Reptin silencing on the repair of DNA double-strand breaks (DSB) in HCC cells. Treatment of HuH7 cells with etoposide (25 μM, 30 min) or γ irradiation (4 Gy) increased the phosphorylation of H2AX by 1.94 ± 0.13 and 2.0 ± 0.02 fold, respectively. These values were significantly reduced by 35 and 65 % after Reptin silencing with inducible shRNA. Irradiation increased the number of BRCA1 (3-fold) and 53BP1 foci (7.5 fold). Depletion of Reptin reduced these values by 62 and 48%, respectively. These defects in activation and/or recruitment of repair proteins were not due to a decreased number of DSBs as measured by the COMET assay. All these results were confirmed in the Hep3B cell line. Protein expression of ATM and DNA-PKcs, the major H2AX kinases, was significantly reduced by 52 and 61 % after Reptin depletion whereas their mRNA level remained unchanged. Phosphorylation of Chk2, another ATM target, was not significantly altered. Using co-immunoprecipitation, we showed an interaction between Reptin and DNA-PKcs. The half-life of newly-synthesized DNA-PKcs was reduced when Reptin was silenced. Finally, depletion of Reptin was synergistic with etoposide or γ irradiation to reduce cell growth and colony formation. In conclusion, Reptin is an important cofactor for the repair of DSBs. Our data, combined with those of the literature suggests that it operates at least in part by regulating the expression of DNA-PKcs by a stabilization mechanism. Overexpression of Reptin in HCC could be a factor of resistance to treatment, consistent with the observed overexpression of Reptin in subgroups of chemo-resistant breast and ovarian cancers.

## Introduction

The ability of tumor cells to repair DNA damage and particularly double strand breaks (DSB) is a major determinant of cancer progression and of resistance to treatment. Understanding the many mechanisms involved is therefore very important in order to find new ways to improve cancer therapy. Reptin (RUVBL2) is a highly conserved essential protein. It belongs to the very large AAA+ (ATPases Associated with various cellular Activities) ATPase family [1,2]. We have previously shown that Reptin is overexpressed in the majority of human hepatocellular carcinomas (HCC) where a high level of expression was associated with a poor prognosis following surgery [3]. We also demonstrated the role of Reptin in HCC progression since its overexpression favored the growth of human xenografts in mice [3], whereas its silencing *in vitro* or in tumors led to cell growth arrest associated with induced senescence [3,4]. An overexpression of Reptin has been reported in several other cancer types like colon [5], stomach [6] and kidney [7] (and see also reviews [8,9]). Similarly, anti-proliferative effects of Reptin silencing have been shown in tumor cells derived from gastric [6] and kidney cancer [7]. Reptin is a pleiotropic protein endowed with many functions relevant to DNA damage repair. For instance, Reptin, together with its homolog protein Pontin, is part of several chromatin remodeling complexes involved at some stage in DNA damage sensing or repair such as INO80 [10], Tip60 [11], Swr1 [12] and YY1 [13]. However, whereas the role of Pontin in DSB repair has been studied in some detail [14], data on the role of Reptin are scarce. Thus in this study, we evaluated the effect of Reptin silencing on the cellular response to DSB in human HCC cells.

## Material and Methods

### Cell culture, siRNA transfection and expression of a Reptin shRNA

The human hepatocellular carcinoma cell lines HuH7 [15] and Hep3B [16] were grown in Dulbecco's modified Eagle's medium (DMEM) supplemented with 10% fetal calf serum in a 5% CO_2_ atmosphere at 37°C. Cells were authenticated using short tandem repeat analysis and tested for mycoplasma contamination on a regular basis. For Reptin silencing, cells were transfected with a siRNA targeting Reptin mRNA, which specificity has been exhaustively tested previously [4]. A siRNA targeting firefly luciferase mRNA, GL2, was used as control. In other experiments, we used cells stably transduced with a lentiviral vector allowing conditional, doxycycline-dependent expression of Reptin shRNA, as described previously [3,17].

### Induction of DNA damage

To induce DNA double-strand breaks, cells were treated with 25 μM *etoposide* (*Sigma Aldrich*) for 30 minutes before analysis, or exposed to 4 Gy gamma radiation (*Gammacell* 3000 *Elan* irradiator) and incubated 2 hours in a 5% CO_2_ atmosphere at 37°C before analysis.

### Flow cytometry analysis

After trypsinization and two washes with Phosphate Buffer Saline (PBS), cells were fixed and permeabilized with the BD Cytofix/Cytoperm buffer (BD Biosciences) for 20 minutes on ice. Cells were then washed with BD Perm/Wash buffer (BD Biosciences) and hybridized for 1 hour in the dark at 4°C with an Alexa 488–conjugated mouse monoclonal anti- γH2AX antibody or an Alexa 488-conjugated mouse IgG1κ isotype control (560445 and 557782, BD Pharmingen) diluted at a ratio of 1:100 in BD Perm/Wash buffer. Cells were then rinsed twice with PBS, and for some experiments stained with DAPI (0.5μg/mL in PBS) during 15 minutes, before analysis on a BD FACSCanto II Flow Cytometer (BD Biosciences).

### Western blot and immunoprecipitation

Cells were lysed in a 1% SDS PBS solution supplemented with protease inhibitor cocktail (Roche). Western blot was done as described previously [17]. All blots were analyzed with the Odyssey system (Li-Cor Biosciences). The following antibodies were used; mouse monoclonal anti-Reptin (BD Biosciences #612482), anti-phospho-Histone H2A.X (Ser139) (Cell Signaling #9718), mouse monoclonal anti-DNA-PKcs (Thermo Scientific #Ab-4), polyclonal rabbit anti-ATM (Abcam #ab10939), rabbit monoclonal anti-phospho-Chk2 (Thr68) (Cell Signaling #2197), mouse monoclonal anti-Chk2 (Cell Signaling #3440), rabbit polyclonal anti-53BP1 (Novus Biologicals #NB100-904), and mouse monoclonal anti-BRCA1 (Santa Cruz s#c6954). After blotting, membranes were stained with SYPRO Ruby Protein Blot Stain (Invitrogen) and scanned with the Molecular Imager PharosFX (Bio-Rad) for the quantification of protein loading. Specific antibody signals were normalized on total protein amount on each entire lane.

For immunoprecipitation, cells were washed in PBS, and lysed in PBS with 1% Triton X-100, Complete x1 (Roche), PhoStop x1 (Roche) and sonicated for 30 seconds on ice. Two milligrams of proteins were incubated overnight at 4°C with 6 μg of the anti-Reptin rabbit polyclonal antibody (ab 137834) from Abcam and 30μL of Dynabeads Protein A (Life Technologies). The beads were washed 4 times with lysis buffer and eluted with Laemmli sample buffer. Eluates were separated on a 3–12% SDS-PAGE for Western blotting.

### Metabolic labeling

This was performed essentially as described previously [17]. Briefly, HuH7 cells were incubated in methionine/cysteine-free medium for 1 h before pulse labeling with 150 μCi/mL EXPRE^35^S^35^S Protein Labeling Mix (Perkin Elmer, Courtaboeuf, France) for 30 min at 37°C. Cells were washed with medium supplemented with 2 mM cysteine/methionine, and cultured for various times in this chase medium. Cells were then scraped in lysis buffer (5 PBS with 1% Triton X-100, Complete x1, Roche) and sonicated for 30 s on ice. Immunoprecipitation was carried out as above. Eluates were separated on a 3–12% SDS-PAGE. The gel was stained with Coomassie blue, destained in water, impregnated with ENHANCE, dried, and exposed to Amersham XP film at -80°C. Autoradiography signals were quantified using Image J [18] and normalized using the intensity of the DNA-PKcs signal on Coomassie Blue staining similarly quantified.

### MTS assay

Cell proliferation assays were performed using tetrazolium compound based CellTiter 96 AQ_ueous_ One Solution Cell Proliferation (MTS) assay (Promega). Cells were exposed to etoposide or gamma radiation at 72 hours after seeding of cells transfected with siRNA targeting Reptin mRNA or the control siGL2 siRNA. MTS assay was performed according to the manufacturer’s instruction at 24, 72, 96 and 120 hours after seeding.

### Clonogenicity assay

Cells were seeded at 800 cells/well in 12-well plates. After 4 days of doxycycline treatment, cells were treated or not with etoposide or irradiated. Following incubation at 37°C for 11 days, the cells were fixed with 1% formaldehyde in ice-cold phosphate-buffered saline (PBS) and stained with 0.05% crystal violet. Colonies with >10 cells were counted under a Olympus IMT-2 microscope.

### Immunofluorescence

Cells were grown on coverslips. Control cells or cells irradiated with 4 Gy or treated with 25μM etoposide were processed 2 hours post-irradiation or 30 minutes after etoposide treatment. Cells were fixed in 4% paraformaldehyde in PBS for 10 minutes at room temperature, permeabilized with 0.1% Triton X100 in PBS and blocked for 30 minutes at 37°C in 4% BSA in PBS. Incubations with anti-53BP1 rabbit polyclonal antibody (1:200, NB100–904, Novus), anti-BRCA1 monoclonal mouse antibody (1:50, sc-6954), or anti-phospho-Histone H2A.X (Ser139) (1:400, 9718, Cell Signaling) and Alexa Fluor 547 donkey anti-rabbit (1:200, FP-SA4000, Molecular Probes) or Alexa Fluor 488 goat anti-mouse (1:200, FP-SA4000, Molecular Probes) secondary antibody were performed for 1 hour at room temperature. Washes were performed in PBS, slides were counterstained with DAPI and eventually mounted in Fluoromount-G (Molecular Probes) before observation and photo capture under an epi-fluorescence microscope (magnification x63). The number of foci was measured using ImageJ using at least 200 cells per experimental condition. High-resolution pictures were taken with a Leica SP5 laser confocal microscope.

### Neutral Comet Assay

Neutral Comet assays were performed with the Trevigen’s CometAssay Kit as described in the manufacturer’s instructions. Cells were analyzed 30 minutes after etoposide treatment or 2h after γ irradiation. Briefly, 400 000 cells were embedded in 1 mL of LMAgarose and 50 μl were deposited on comet slides. The slides were incubated 1 hour at 4°C in lysis solution, then immersed in freshly prepared neutral electrophoresis buffer (Tris Base 1 M, sodium acetate 3 M, pH 9.0) for 30 minutes at 4°C in the dark. Electrophoresis was carried out for 1 hour at 20 V in electrophoresis solution. Slides were then stained with SYBR Green I. DNA strand breakage was expressed as “comet tail moment”. Tail moment was measured for at least 100 cells per sample. Tail DNA content was analyzed with the CometScore software (TriTek Corp).

### RT—qPCR

Real time quantitative PCR was performed using the StepOnePlus Real-Time PCR System (Applied Biosystems) with B-R SYBR Green SuperMix for iQ (Quanta Biosciences). Primer used were as follows. For DNA-PKcs, forward 5’-AAAATTGGTACCCCCGACAG-3’, reverse 5’-TTCTCATGACCCAGGAGTAGC-3’; for ATM, forward 5’-TGCTGACAATCATCACCAAGTTC-3’, reverse 5’-TCTCCCTTCGTGTCCTGGAA-3’. The specificity of the amplification reactions was monitored by melting curve analysis. The threshold cycle (*C*_t_) value for each gene was normalized to the *C*_t_ value for RNA18S5 and all relative levels of expression (2^-ΔΔCt^) were calculated [19]. All samples were analyzed at least in triplicate.

## Results

**Fig 1:**
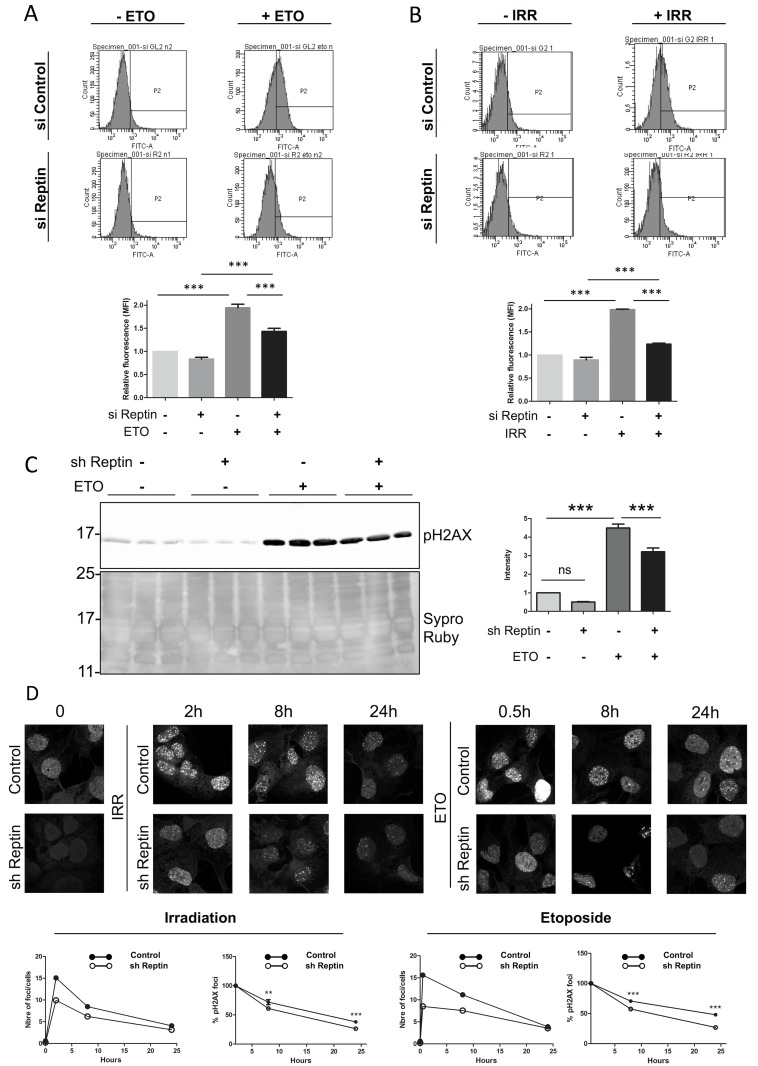
Reptin depletion impairs H2AX phosphorylation at serine 139 (γH2AX) after DNA damage. (A) and (B) HuH7 cells were transfected with a control or a Reptin siRNA. After 3 days, they were then treated with etoposide (ETO, 25 μM) (A) or exposed to gamma radiation (IRR, 4 Gy) (B), stained for γ-H2AX and analyzed by flow cytometry. The bar graphs show the mean of 3 experiments (*** p<0.001 by One-way ANOVA followed by Bonferroni test). (C) and (D) HuH7 cells stably expressing a doxycycline-inducible Reptin shRNA were treated with doxycycline (sh Reptin +) or left untreated (sh Reptin-) for 4 days. Extracts from HuH7 cells treated with etoposide as in (A) were analyzed by Western blot with an anti-phospho-H2AX antibody (C). Data were normalized relative to the Sypro Ruby staining of the membrane. The graph on the right shows the mean of 9 experiments (*** p<0.001 by One-way ANOVA followed by Bonferroni test). (D) Control or Reptin depleted HuH7 cells were treated with etoposide or irradiated, then fixed at different time points and immunostained for phospho-H2AX. The graphs below show for each treatment the evolution of the number of foci per cell (left) or the fractional decrease of the number of foci, setting the initial time point as 100% (right).

In a first series of experiments, DNA DSBs were induced by treatment of cells with etoposide. Phosphorylation of H2AX on Ser139, which is one of the earliest events following DSB induction, was used as readout and was analyzed by flow cytometry. These experiments were carried out in the human HCC cell line HuH7 transfected with an anti-Reptin siRNA, Etoposide treatment induced a 1.94 ± 0.13 fold increase in the phosphorylation of H2AX Reptin depletion by itself had no effect on H2AX phosphorylation. However, H2AX phosphorylation following etoposide was decreased by 35.5% ± 0.2% (p<0.001) when Reptin was depleted (Fig 1A), and this was identical when analyzed with Western blot (Fig 1C). Similar results were obtained when DSBs were induced by γ irradiation. H2AX phosphorylation was significantly stimulated by 2.0 ± 0.02 fold by irradiation, but Reptin depletion reduced the stimulation by 65.5 ± 7.1% (p<0.001) (Fig 1B). FACS analysis showed similar results in another HCC cell line, Hep3B (S1 Fig). Finally, the changes in H2AX phosphorylation following etoposide or γ-irradiation were independently confirmed in both HuH7 and Hep3B cells using immunofluorescence and fluorescent foci quantitative analysis (Fig 1D and S2 Fig). In this experiment, we also followed the kinetics of the disappearance of γH2AX foci. As shown on Fig 1C and S2 Fig, at every time point, H2AX phosphorylation was decreased in Reptin-depleted cells as compared to control cells. In addition, the disappearance of the foci, as assessed by the change in the percentage of cells with foci, was significantly slower when Reptin was silenced. This was shown for HuH7 cells for both etoposide and irradiation (Fig 1D). For Hep3B cells, it was significant for etoposide although there was only a trend after irradiation (S2 Fig).

Because cell sensitivity to DNA damaging agents may vary according to the cell cycle status, with cells in G1 being less sensitive [20–22], we checked how Reptin silencing affected cell cycle progression. We found that Reptin silencing had no dramatic effect on cell cycle progression as assessed by flow cytometry following DNA labeling. There was no significant change in HuH7 (S3 Fig), and a small but significant reduction of cells in G1 in Hep3B cells, at the expense of an increase in cells in G2/M (S3 Fig). In addition, double staining with DAPI and anti- γH2AX showed that the majority of γH2AX-positive cells were in the G2/M phase in both control and Reptin-depleted cells, with no significant difference between the 2 conditions (S3 Fig).

**Fig 2:**
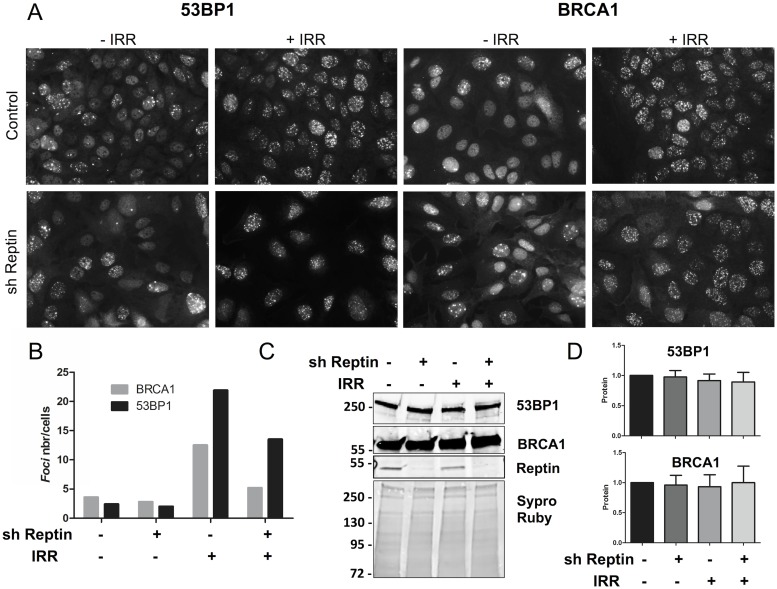
Reptin depletion reduces the recruitment on chromatin of BRCA1 and 53BP1 after gamma ray irradiation. HuH7 cells stably expressing a doxycycline-inducible Reptin shRNA were treated with doxycycline (sh Reptin +) or left untreated (sh Reptin-) for 4 days. (A) Representative images of 53BP1 and BRCA1 foci in HuH7 cells detected using immunofluorescence 2h after gamma ray irradiation. (B) The bars represent the mean number of foci per cell from two independent experiments (>200 cells were counted per experiment). Expression levels of BRCA1 and 53BP1 were assessed by Western Blot on whole cell extracts 4 days after doxycycline treatment. A representative picture is shown in (C). The migration positions of molecular weight standards (in kDa) are indicated on the left. (D) Quantification of 3 Western blot experiments.

Phosphorylated H2AX serves as a platform for the ordered recruitment of many proteins that participate to DNA repair. We thus followed the recruitment on chromatin of two such key proteins, BRCA1 and 53BP1. This was done using immunofluorescence and image analysis on HuH7 cells undergoing γ irradiation. Irradiation induced a 3-fold increase in the number of BRCA1 foci and a 7.5 fold increase in the number of 53BP1 foci. Reptin depletion decreased these values by 62 and 48%, respectively. Total levels of BRCA1 and 53BP1 were not changed upon Reptin depletion, as shown by Western blot (Fig 2). We also used immunofluorescence to follow the kinetics of disappearance of 53BP1 foci in HuH7 and Hep3B cells. As for γH2AX kinetics, there were less 53BP1 foci at every time point following DNA damage upon Reptin silencing (S4 Fig).

**Fig 3:**
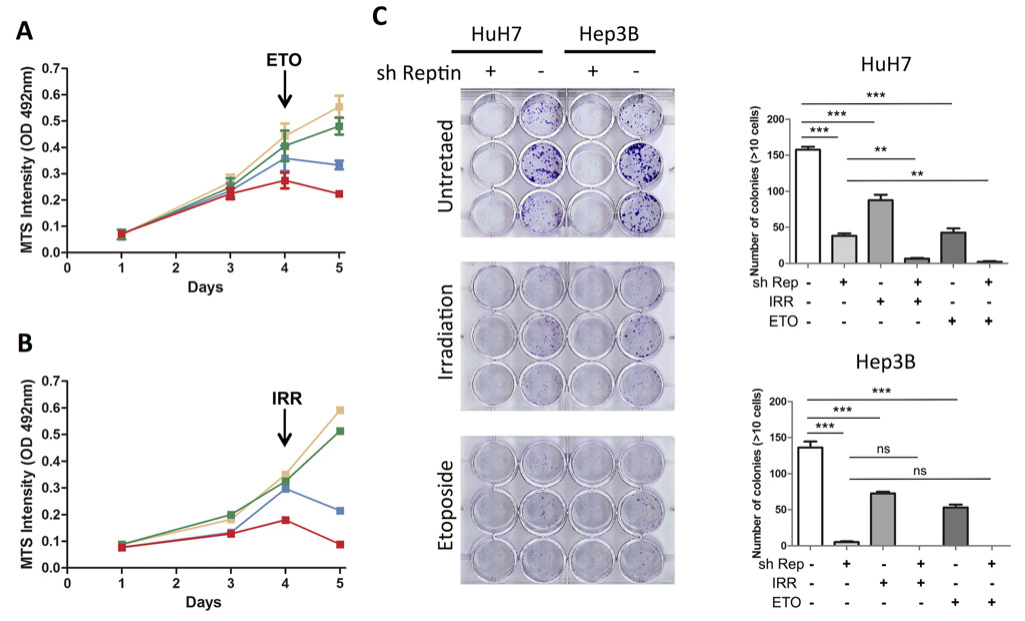
Reptin depletion and DNA damage cooperate to reduce cell viability. (A) HuH7 cells transfected with a control siRNA were either left untreated (yellow) or received etoposide (ETO, green) after 3 days. Similarly, cells transfected with an anti-Reptin siRNA (siR2) were untreated (blue) or received etoposide (red). The effect on cell number was determined at 1, 3, 4 and 5 days after seeding, using the MTS assay. The results are the mean ± SD of 3 independent experiments. After 5 days, the difference between siR2 and siControl without treatment was significant with a p value <0.001 and the difference between untreated siR2 and treated siR2 was significant with a p value <0.05 (Two-way ANOVA followed by Bonferroni test). (B) Same design as in (A) except that DNA damage was induced with γ-irradiation (IRR). The figure shows the mean of 2 independent experiments. (C) Clonogenic tests were carried out as described in Materials and Methods using HuH7 cells stably expressing a doxycycline-inducible Reptin shRNA that were either treated with doxycycline (sh Rep +) or untreated (sh Rep-). The graph shows the mean of 3 replicates (*** p<0.001 and ** p<0.01 by One-way ANOVA followed by Bonferroni test).

In order to analyze the functional consequences of the defective DSB signaling in Reptin-depleted cells, we analyzed cell viability following γ irradiation, first using the MTS assay. Four days after transfection with the anti-Reptin siRNA, depletion of Reptin, or γ irradiation alone, led to a slight decrease in cell growth. However, the combination of γ irradiation and Reptin depletion was additive to reduce cell growth (Fig 3A). Similar results were obtained when combining etoposide and Reptin depletion (Fig 3B). We further confirmed this result using a colony formation assay. As shown on Fig 3C, silencing Reptin, etoposide treatment or γ irradiation all led to a significant impairment in colony formation in HuH7 cells. However, combining Reptin depletion with DNA damage further reduced clonogenicity. A similar trend was seen with Hep3B cells but was not statistically significant because the effect of Reptin depletion alone was already very strong.

**Fig 4:**
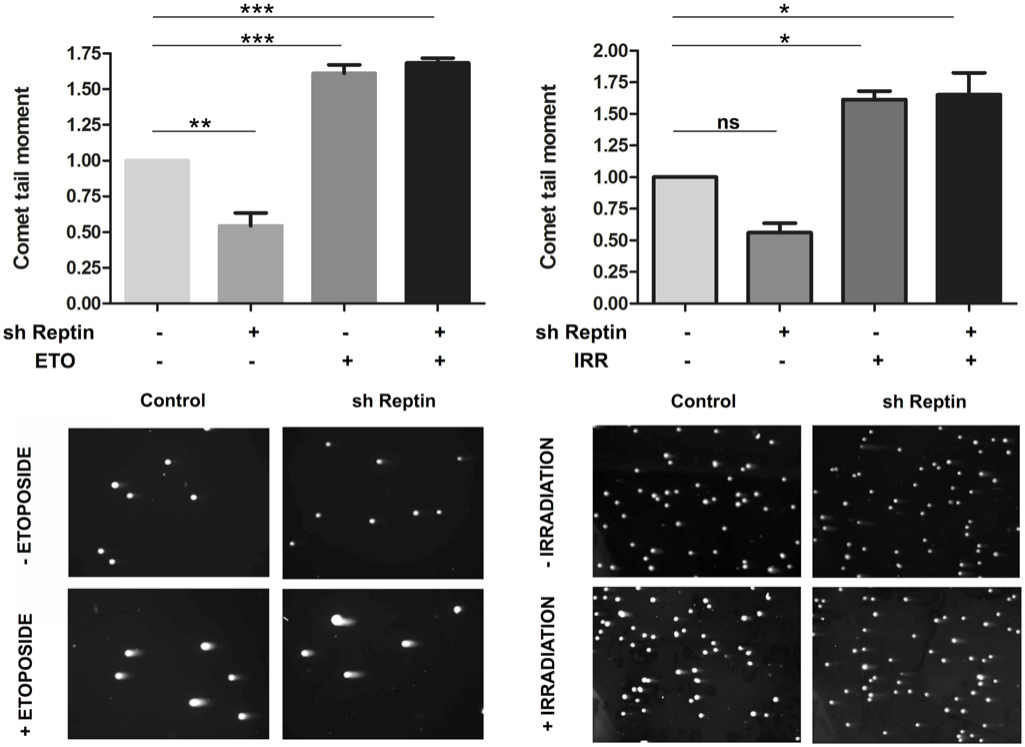
Reptin depletion does not reduce the number of double strand breaks. HuH7 cells were treated as in Fig 1. The neutral COMET assay was used to assess DNA double-strand breaks that were quantified using calculation of the comet tail moment. Bars represent the mean ±SD from 3 independent experiments (>140 cells were counted per experiment). Left, etoposide treatment, right, γ irradiation. * p<0.05, ** p<0.01 and *** p<0.001 by One-way ANOVA followed by Bonferroni test. Representative COMET images are shown below.

We reasoned that the defective phosphorylation of H2AX, and subsequent recruitment of BRCA1 and 53BP1, might be due to a decreased sensitivity of Reptin-depleted cells to DNA damage. We thus quantified the number of DSB following etoposide or γ irradiation treatment, using the COMET assay. Unexpectedly, depletion of Reptin reduced the number of basal DSB in the absence of DNA damage induction, as assessed by COMET tail moment measurement. However, treatment with etoposide or γ irradiation induced the same amount of DSB whether Reptin was depleted or not (Fig 4).

**Fig 5:**
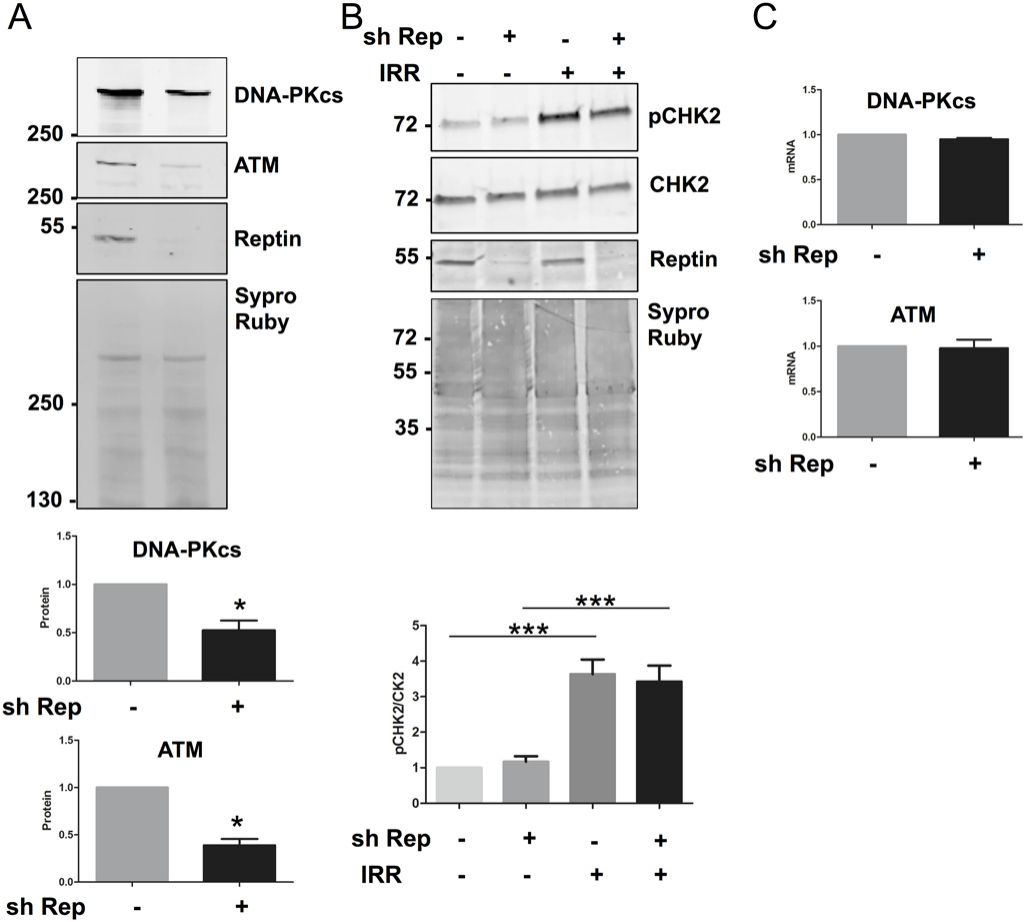
Effect of Reptin depletion on signaling intermediates upstream of H2AX phosphorylation. (A) Protein expression of DNA-PKcs and ATM after Reptin depletion were analyzed by Western Blot on whole cell extracts 4 days after Reptin silencing with doxycycline (sh Rep +) (n = 6 and n = 3 for DNA-PKcs and ATM, respectively: * p<0.05 by Mann and Whitney test) (B) Phosphorylation of CHK2 after Reptin depletion and 2h after gamma ray irradiation (+ IRR) was detected by Western Blot and normalized on total CHK2 (n = 4: *** p<0.001 by One-way ANOVA followed by Bonferroni test) (C) RNA was extracted 4 days after Reptin silencing and expression of DNA-PKcs and ATM mRNAs was analyzed by RT-qPCR. (n = 3).

We thus hypothesized that H2AX phosphorylation could be reduced because of an upstream signaling defect. H2AX phosphorylation following DNA DSB is mainly performed by DNA-PKcs and ATM (reviewed in [23]). Expression of DNA-PKcs and ATM proteins, assessed by Western blot, was decreased by 52 ± 25% (p = 0.05) and 61 ± 12% (p = 0.01) when Reptin was depleted (Fig 5A). Phosphorylation on Thr68 of Chk2, another ATM substrate, was significantly enhanced following irradiation (x 3.63 ± 0.82% p<0.001), but its phosphorylation remained identical in cells with Reptin depletion, suggesting that although ATM expression is reduced, its level is not limiting (Fig 5B).

**Fig 6:**
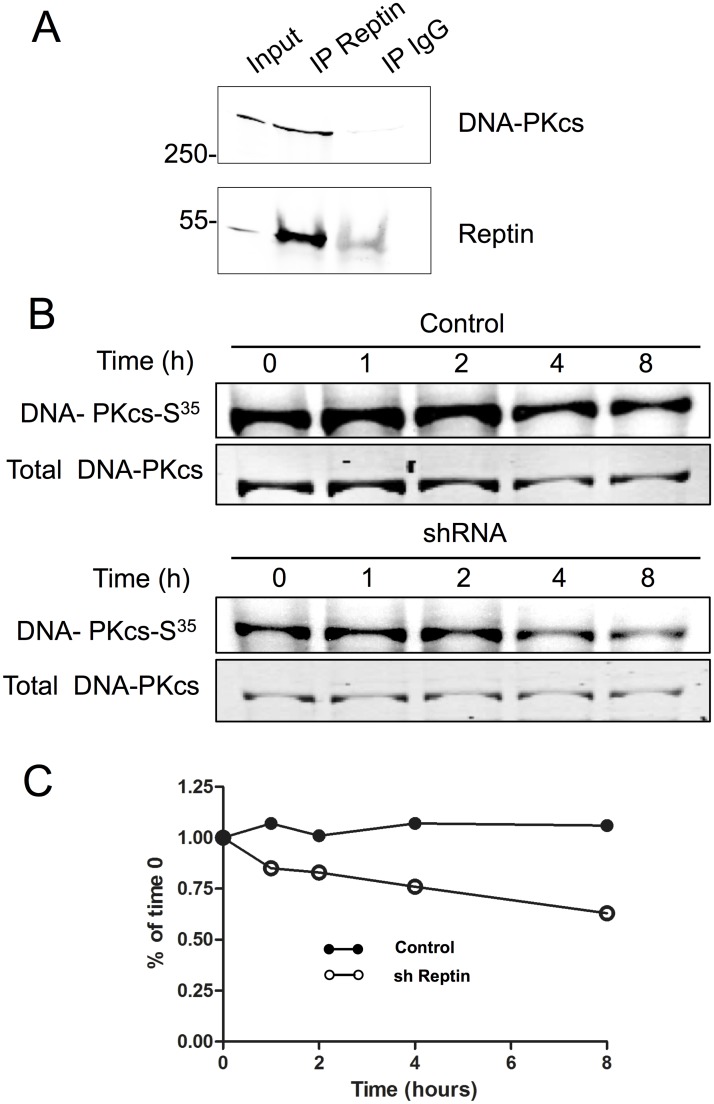
Reptin interacts with DNA-PKcs and regulates its stability. (A) Interaction between Reptin and DNA-PKcs was tested by immunoprecipitation. The migration positions of molecular weight standards (in kDa) are indicated on the left. The faint band seen in the IgG lane with the Reptin antibody corresponds to traces of IgG heavy chains. The picture is representative of 3 similar experiments. (B) Metabolic labeling and pulse chase. HuH7 cells stably expressing a doxycycline-inducible Reptin shRNA were treated (sh Reptin) or not (Control) with doxycycline. After 4 days, they were labeled with EXPRE^35^S^35^S as described in Materials and Methods. Following the indicated periods of chase, DNA-PKcs was immunoprecipitated and the eluates were separated on SDS-PAGE. The top panel shows the autoradiographic image, and the bottom one the Coomassie blue staining of the gel with DNA-PKcs. (C) The graph shows the quantitative analysis of the data following normalization of the autoradiographic signal on the amount of immunoprecipitated DNA-PKcs.

The mRNA levels of DNA-PKcs and ATM were unchanged when Reptin was depleted, indicating that decreased protein levels were due to a post-transcriptional regulation (Fig 5C). Since it was previously shown that Reptin and DNA-PKcs could interact in several cell types [24], we immunoprecipitated Reptin from cell extracts and demonstrated the co-precipitation of DNA-PKcs by immunoblot (Fig 6A). Conversely, when DNA-PKcs was immunoprecipitated, Reptin could be detected in the precipitate using label-free mass spectrometry (not shown). We then analyzed DNA-PKcs stability by treating control cells or cells with Reptin silencing (4 days after induction of shRNA) with cycloheximide and measuring the residual amount of DNA-PKcs over time. These experiments showed that although the initial level of DNA-PKcs protein expression was lower in Reptin depleted cells, the stability of the pool of protein was the same in control and Reptin depleted conditions, the protein being extremely stable (S5 Fig). We thus performed metabolic labeling and pulse chase experiments to investigate the stability of newly synthesized DNA-PKcs. We found that in control cells, newly synthesized DNA-PKcs remained completely stable for at least 8 hours. On the other hand, when Reptin was silenced, DNA-PKcs was clearly destabilized (Fig 6B).

## Discussion

The AAA+ ATPase Reptin has been found overexpressed in many cancers (reviewed in [9]) including hepatocellular carcinoma and kidney cancer where overexpression was associated with a poor prognosis [3,7]. Reptin is present in several protein complexes including Tip60 that is a major actor of DNA damage repair [11]. We thus investigated whether Reptin could regulate the response to DNA damage in HCC cells. We show here that Reptin is indeed involved in the repair of DNA DSBs in human HCC cells. RNAi-induced Reptin depletion led to a decreased phosphorylation of H2AX and subsequent recruitment of BRCA1 and 53BP1. DNA damage repair was also likely altered as seen with the slower disappearance of γH2AX from the chromatin. Ultimately, the altered DNA damage response was associated with decreased cell viability and ability to form colonies. Similar results were observed following two different types of DNA damaging and in both HuH7 and Hep3B cells. Hep3B cells were however more sensitive to the effect of Reptin silencing than HuH7 cells, which may be because the latter lack functional p53 protein [25]. We and others have previously shown that Reptin silencing by itself reduced tumor cell growth [3,4,7,26,27]. It is thus likely that defective DNA repair could contribute to this effect and lead to the dramatic senescence observed upon Reptin silencing in liver or kidney cancer cells [4,7,28].

A few studies have addressed the role of Reptin in the DNA damage response in tumor cells and led to different results. Thus Reptin depletion did not change the amount of γH2AX following γ irradiation in prostate PC3 cells, although this was not precisely quantified [29]. On the other hand, Reptin depletion did not decrease but rather enhanced the γH2AX response to UV irradiation in HeLa cells [30]. This may represent a difference because of the nature of the DNA lesions or a cell type specific feature. There are also a few data in non-tumor cells. Thus, the normal human fibroblast cells MRC5 showed an hyperphosphorylation of H2AX upon γ irradiation when Reptin was silenced [31] whereas mutant zebrafish that have an undetectable expression of Reptin exhibit a spontaneous hyperphosphorylation of H2AX in the brain [32]. There may thus be differences between tumor and non-transformed cells where Reptin is expressed at lower levels.

Since Reptin silencing did not alter the number of DSBs, we hypothesized that the decreased γH2AX response was consecutive to a decreased activity of the kinases that phosphorylate it. Upon DSB induction, H2AX is mostly phosphorylated by DNA-PKcs and ATM, two members of the PIKK family [23]. We found that the total level of these kinases was decreased by more than 50% when Reptin was silenced. We further suggest that although both DNA-PKcs and ATM levels were similarly reduced, the decreased γH2AX level may be preferentially related to the loss of DNA-PKcs since the phosphorylation of Chk2, another ATM substrate, was unaffected, suggesting that ATM amount is not limiting under these conditions. It is noteworthy that Reptin silencing did not either modify Chk2 phosphorylation following UV irradiation in HeLa cells [30].

The decreased expression of DNA-PKcs and ATM upon Reptin silencing was already reported in HeLa cells [24,33]. However, in these studies, decreased levels of DNA-PKcs and ATM were associated with a reduction of their respective mRNAs, suggesting that at least part of the regulation was transcriptional. Here, we show that the levels of the transcripts of these proteins are not affected by Reptin depletion, showing a strictly post-transcriptional regulation. This is supported by the finding that Reptin and DNA-PKcs do interact, as shown by co-immunoprecipitation, confirming previous studies [24]. We further demonstrate that Reptin silencing results in a reduced stability of DNA-PKcs. The combination of experiments of pulse chase and of western blot following cycloheximide show that Reptin silencing specifically affects the half-life of newly synthesized DNA-PKcs, but has no effect on the stability of the pre-formed pool of the protein. These results are in coherence with the participation of Reptin and its homolog Pontin to the R2TP complex. This complex, initially isolated in yeast [34], is composed of Rvb1 (yeast Pontin), Rvb2 (yeast Reptin), Tah1 (RPAP3 in human) and Pih1D1 (or NOP17) and is highly conserved in Drosophila [35], Apicomplexa [36] and human [37]. It is a co-chaperone of HSP90 and is involved in the stabilization of every PIKK family member through the Tel2 adaptor protein [38–40]. It was shown that Tel2 interacts with PIKK proteins only during the first hour after their synthesis [39], and that Tel2 silencing resulted in decreased stability of newly synthesized mTOR and ATM [38]. This is however the first time that the mechanism for Reptin silencing induced DNA-PKcs down-regulation is defined. On the other hand, the lack of effect of Reptin silencing on the stability of the pool of DNA-PKcs is in agreement with data showing no apparent changes in DNA-PKcs stability in cells silenced for Pontin and treated with cycloheximide [24], and with reports showing a very long half-life for DNA-PKcs, up to more than 5 days [41].

Altogether, we thus propose that Reptin, mostly via the post-transcriptional stabilization of DNA-PKcs, controls the signalization of DNA damage. Reptin depletion leads to a decrease in DNA-PKcs levels, with consecutive reduced phosphorylation of H2AX and defective repair of lesions. Conversely, it can be hypothesized that the high Reptin levels observed in many cancers could confer a higher ability of tumor cells to repair the DNA damage and thus contribute to their resistance to treatment. This would fit nicely with the overexpression of Reptin observed in ovarian cancers resistant to platin-based chemotherapy [42] and in chemoresistant breast cancers [43].

## Supporting Information

S1 Fig Reptin depletion impairs H2AX phosphorylation at serine 139 (γH2AX) after DNA damage in Hep3B cells. (TIF)

S2 Fig Kinetics of H2AX phosphorylation following DNA damage in Hep3B cells. (TIF)

S3 Fig Cell cycle analysis. (TIF)

S4 Fig Kinetics of 53BP1 foci analyzed by immunofluorescence. (TIF)

S5 Fig Effect of cycloheximide on the stability of DNA-PKcs. (TIF)
