# Supplementary figures and images for: Reptin Regulates DNA Double Strand Breaks Repair in Human Hepatocellular Carcinoma
Source: PLoS One. 2015 Apr 15;10(4):e0123333. doi: 10.1371/journal.pone.0123333 (PMC4398330; doi:10.1371/journal.pone.0123333)

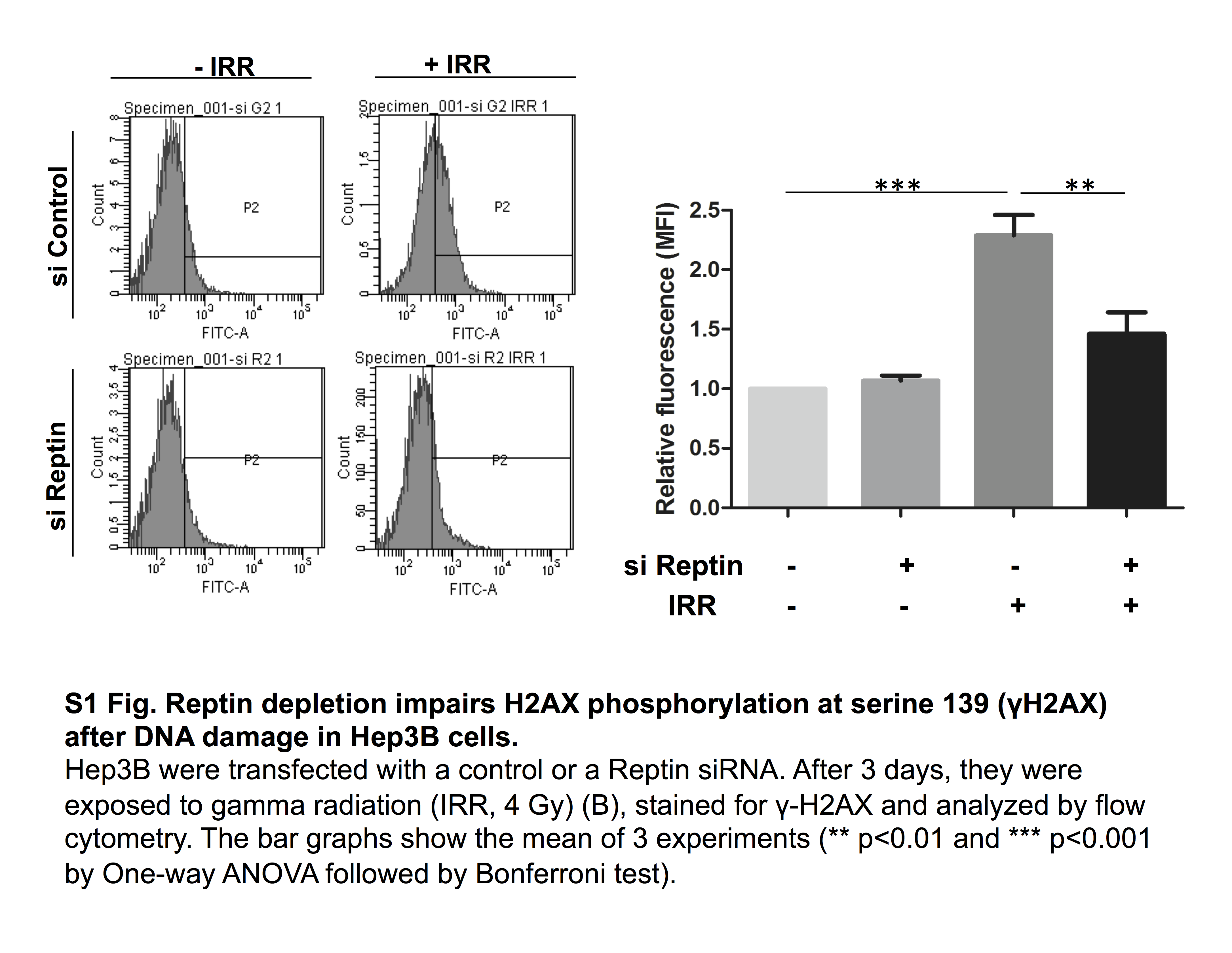

Supplement: S1 Fig — (TIF) [file pone.0123333.s001.tif]

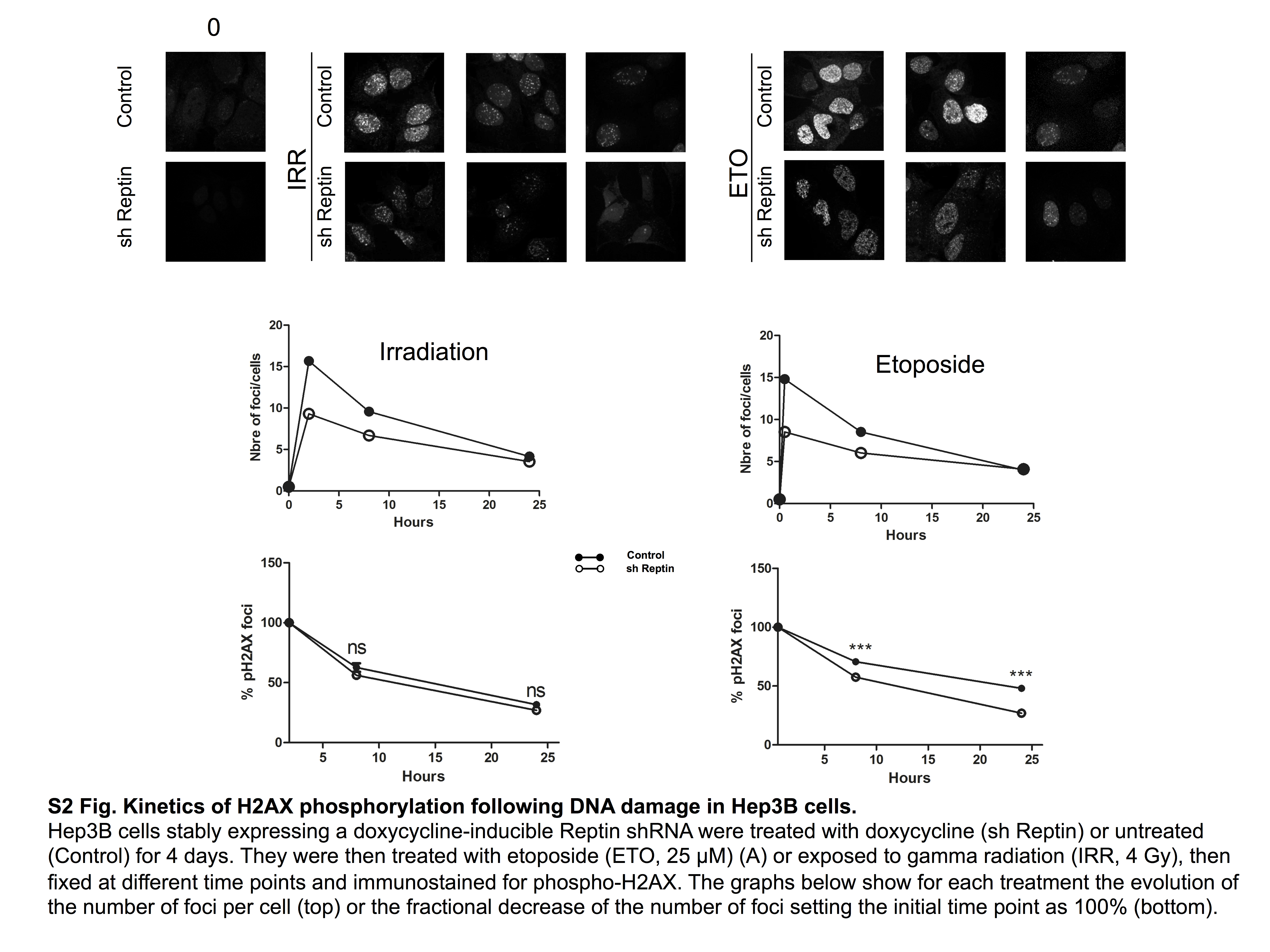

Supplement: S2 Fig — (TIF) [file pone.0123333.s002.tif]

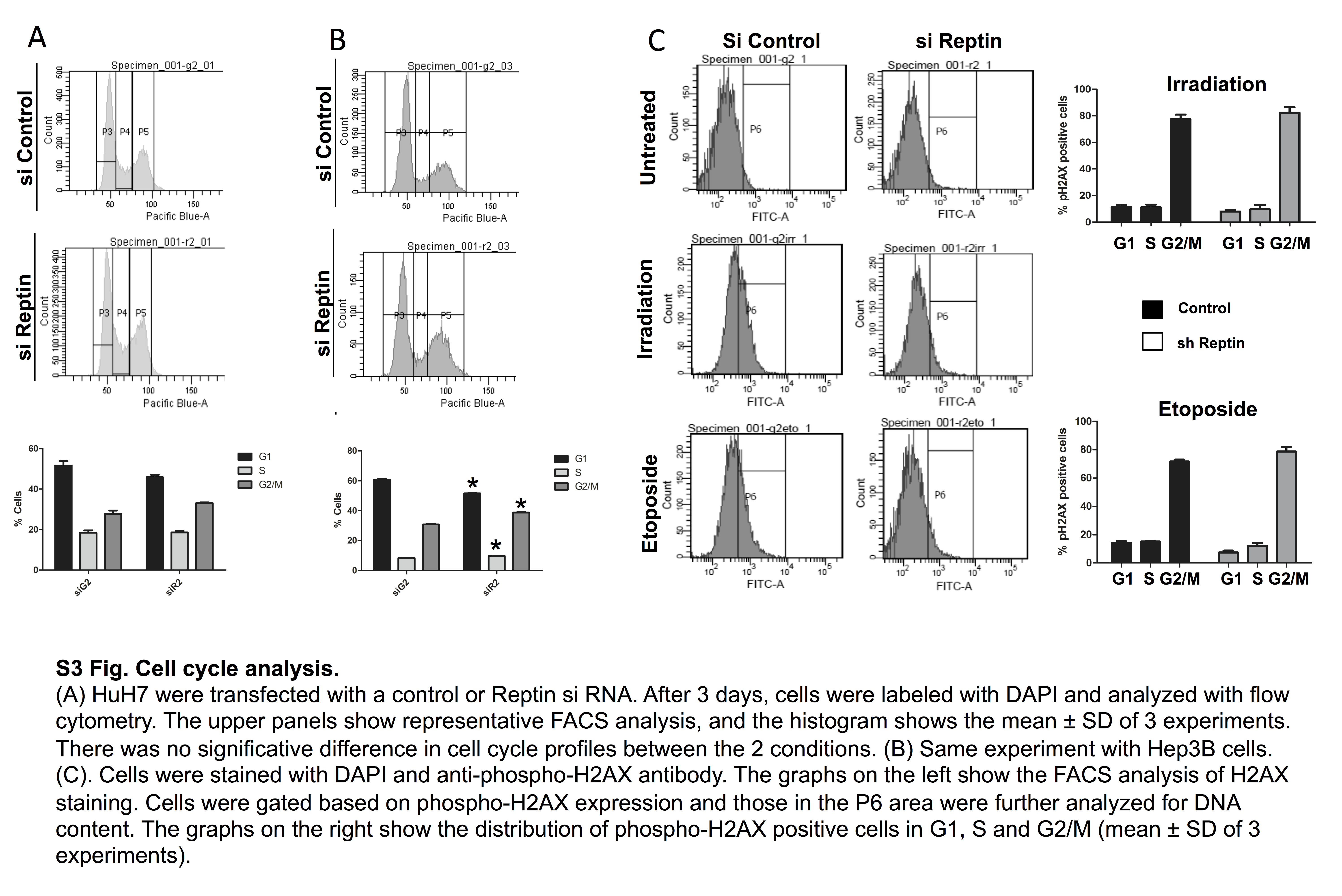

Supplement: S3 Fig — (TIF) [file pone.0123333.s003.tif]

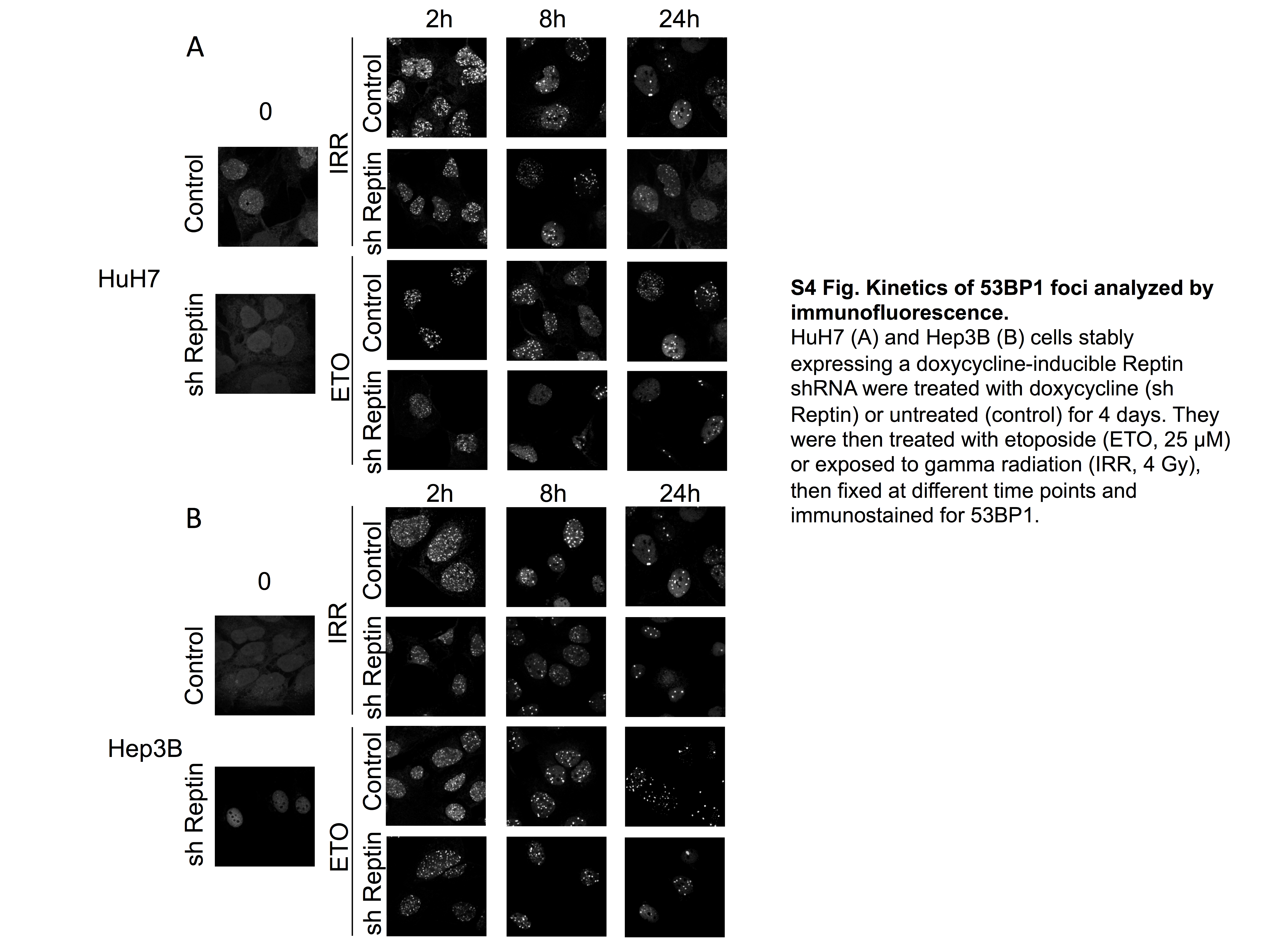

Supplement: S4 Fig — (TIF) [file pone.0123333.s004.tif]

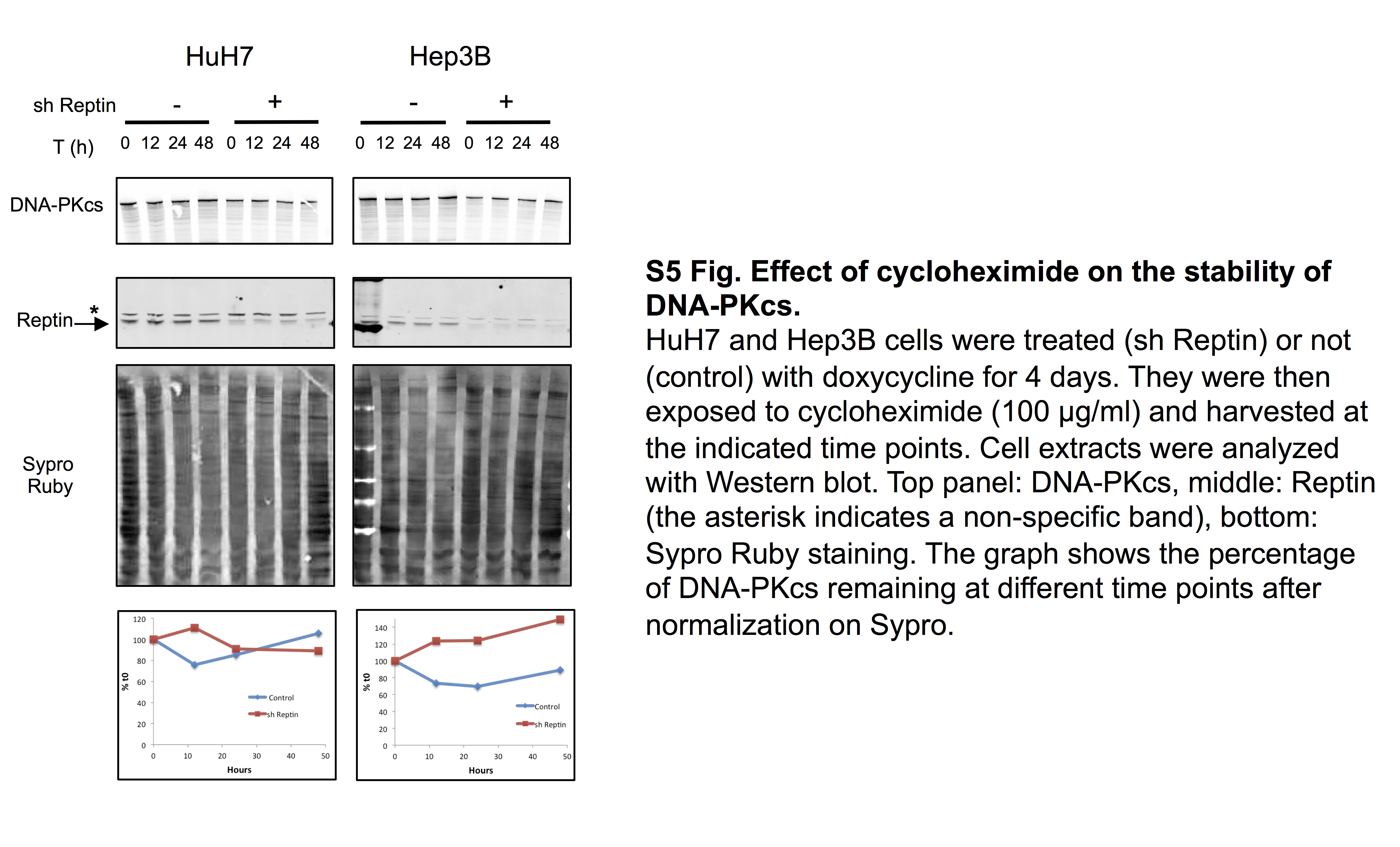

Supplement: S5 Fig — (TIF) [file pone.0123333.s005.tif]
